# Which Fishers are Satisfied in the Caribbean? A Comparative Analysis of Job Satisfaction Among Caribbean Lobster Fishers

**DOI:** 10.1007/s11205-012-0058-0

**Published:** 2012-05-16

**Authors:** Iris Monnereau, Richard Pollnac

**Affiliations:** 1 Centre for Maritime Research (MARE), University of Amsterdam, Amsterdam, The Netherlands; 2 Marine Affairs, University of Rhode Island, Kingston, RI 02881 USA

**Keywords:** Job satisfaction, Caribbean, Lobster fisheries, Governance

## Abstract

Lobster fishing (targeting the spiny lobster *Panulirus argus*) is an important economic activity throughout the Wider Caribbean Region both as a source of income and employment for the local population as well as foreign exchange for national governments. Due to the high unit prices of the product, international lobster trade provides a way to improve the livelihoods of fisheries-dependent populations. The specie harvested is identical throughout the region and end market prices are roughly similar. In this paper we wish to investigate to which extent lobster fishers’ job satisfaction differs in three countries in the Caribbean and how these differences can be explained by looking at the national governance arrangements.

## Introduction

A variety of researchers have carried out job satisfaction studies in North American fisheries during the last decades (Pollnac and Poggie 2006; Binkley 1995; Gatewood and McCay 1988, 1990). The significant role of self-actualization in the determination of job satisfaction has been one of the key outcomes in all these studies. This backs the reasoning that fishing is more than just a livelihood, but rather ‘a way of life’ (Gatewood and Mccay 1988: 126). Pollnac et al. (2001) make a similar point for South-East Asian fishers and therefore argue that contrary to the expectation of fisheries managers, fishermen are not likely to be interested in alternative employment (see also Binkley 1995; Gatewood and McCay 1990; Pollnac and Poggie 1988). McGoodwin claimed that even when fishers face declining catches and falling incomes they will still hold onto their occupation (1990). Smith (1981) even found evidence in the North American pacific salmon fishery where 70 % are willing to subsidize their fishing with money earned through other pursuits. This could relate to the research in industrialized countries indicating that fishers derive many types of satisfaction from their occupation and resist changes that would reduce this satisfaction (Binkley 1995; Gatewood and McCay 1990; Pollnac and Poggie 1988).

Job satisfaction analysis is an attractive method as it facilitates comparing fisher’s satisfaction both within as well as between countries. The survey applied uses five different categories of job satisfaction; *basic needs*, *social needs*, *self*-*actualization*, *management* and *nature*. This study aims to investigate differences in job satisfaction between lobster fishing populations in the Caribbean, focusing on Belize, Nicaragua and Jamaica.

Lobster fishing (targeting the spiny lobster *Panulirus argus*) is an important economic activity throughout the Wider Caribbean Region, both as a source of income and employment for the local population as well as foreign exchange for national governments. At present 50,000 lobster fishers are estimated to be active in the Wider Caribbean Region, with an additional 200,000 people working in positions related to the lobster fishery (FAO 2003). An average of 27–30 thousand tons per annum are caught in the Wider Caribbean region annually with a corresponding value of around 500 million US$ (Cochrane and Chakallal 2001). Landings peaked during 1987–1997 at about 37–43 thousand metric tons, but regional landings decreased 55 % in the 2000s (Ehrhardt et al. 2011). This is mainly due to intensive exploitation and to environmental and ecological changes in the spiny lobster habitat (ibid.). High demand and reduced supply significantly increased prices paid for lobster until 2007. Importers’ prices paid per pound increased tremendously until they were paying around 21–22 US per pound. Since 2008 however, the economic crisis has severely affected the high unit prices of lobster. As consumer demand in North America for the luxury product vanished, prices paid per pound for lobster dropped as much as 33 % (Monnereau and Helmsing 2011). Since 2010 the prices have been climbing again (ibid.).

Due to the high unit prices of the product previous to the crisis and the magnitude of production, the trade provides a way to improve the livelihoods of fisheries-dependent populations in the Wider Caribbean Region. The lobster value chain, running from the moment the lobster is harvested until it reaches the consumer, often in the United States, shows many similarities between the three countries. Nevertheless the national governance arrangements differ substantially from one another in each country. In this paper we investigate to what extent fishers’ job satisfactions are related to these differences and to what extent they are considering changing their professions.

## Methodology

The survey of job satisfaction used in the current research is based on the model developed by Pollnac and Poggie (1988), to which two categories of questions have been added. The survey therefore consists of five categories: basic needs, social needs, self-actualization, management and the value of nature. The *basic needs* category relates to fishers’ health, earnings, and their ability to feed their family. The second category of social needs refers to fishers’ satisfaction with time at sea, being one’s own boss and the time they spend away from their families. *Self*-*actualization* relates to the notion of fishing as a challenging, adventurous and worthwhile occupation. Management is new category and considers views on conflict and conflict resolution, rules and regulations, performance of government officials, possibilities for participation, and overall management. The nature category refers to the satisfaction of fishers with their landing site as well as level of fish stocks.

The research survey was administered to a sample of 83 lobster fishers from Belize, Jamaica and Nicaragua. Thirty-one fishers were interviewed in Belize, 26 in Jamaica and 26 in Nicaragua. Despite the fact that fishers are divided by gear type (trap fishers as well as divers) the majority of respondents are small-scale. Only 4 respondents in Nicaragua employ industrial type methods. The industrial trapping boats are steel vessels with large freezer holds that go out to sea for 45 days at a time.

The Likert scale was used to rank the responses to the 27 questions in the survey. When responding to a Likert questionnaire item, fishers specify their level of agreement according to a five-point scale ranging from (1) very unsatisfied, (2) unsatisfied, (3) neutral, to (4) satisfied and (5) very satisfied. In addition three overall questions on job satisfaction were added relating to whether a fisher would enter a job outside of fishing, move to another type of fishing and whether he or she would advise a young adult to enter the fishery.

In Jamaica the survey was administered to a sample of 26 trap fishers and divers in six communities: Crawford, Black River, Alligator Pond, Whitehouse, Gallon beach, and Treasure Beach. These communities are located in Westmoreland parish. Westmoreland is one of four parishes with the highest production of lobster in Jamaica. Not all the fishers limit their activities to the inshore zone; some go for 8–9-day fishing trips to Pedro Bank, located some 80 km away from the mainland. The researchers approached fishers randomly at the landing beaches where they would arrive in the afternoon (in the case of day fishers) and in the night/morning (in the case of Pedro Bank fishers).

In Nicaragua, the survey was carried out on Corn Island. Corn Island is responsible for half of the lobster landings in Nicaragua. Three fishing groups are available on the island: small-scale divers, small-scale trappers and industrial trappers. The industrial diving fleet, which is also of importance in the northern zone of Nicaragua, is not present in this area and therefore not represented in the survey. The researchers administered the survey to a sample of 26 fishers, 18 small-scale trap fishers, 4 industrial trap fishers and 4 small-scale divers. The researchers interviewed a random sample at the different lobster buying locations where fishers would sell their catch in the afternoon, or at the central ‘park’ on the island. Industrial fishers were approached at the piers of processing plants and the public pier where the industrial boats are stationed.

**Fig. 1:**
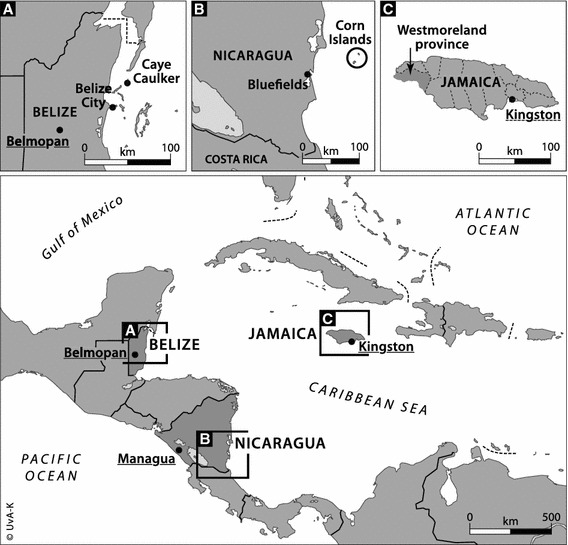
Current differences between the countries. Location of research sites in Jamaica, Nicaragua and Belize (*Source*: UvA Kaartenmakers)

In Belize, the survey was administered to a sample of trap and dive fishers in two locations. Twenty fishers were interviewed in Caye Caulker and eleven in Belize City. All lobster divers are stationed in Belize City. Caye Caulker is one of four coastal communities where day fishers use traps to catch lobster. A small cooperative associated with one of the big fishing cooperatives in Belize City is based in Caye Caulker. In both locations, the researchers approached fishers at the landing site (Fig. 1).

Table 1 indicates that Nicaragua is the poorest of the three countries and holds the 115th place on the UN’s Human Development Index (UNDP 2010). Jamaica and Belize hold similar ranks at respectively the 80th and 78th position (UNDP 2010). The table also shows that in Belize, the contribution of the fishing sector to the national GDP is the highest of all three. In Belize it’s 5 %, in Jamaica and Nicaragua only respectively 0.5 and 0.11 %.

**Table 1:** General characteristics of Jamaica, Nicaragua, Belize

	Jamaica	Nicaragua	Belize
Area (km^2^) (thousands)	11.0	130.0	23.0
EEZ (km^2^)	274.00^a^	110.922^b^	169.840^b^
% of GDP fishing sector	0.5^d^	0.11^e^	5^f^
Human Development Index^g^	0.0688 (80th place)	0.565 (115th place)	0.694 (78rd place)

*Sources*: World Development Indicators database, April 2009. Indicators based on 2008 data. Except for when indicated below

^a^FAO Fisheries and Aquaculture Country Profiles: Jamaica and Nicaragua. Accessed on 3 November 2009

^b^Ibid

^c^Caribbean Regional Fisheries Mechanism (CRFM). Quick facts: Belize. Accessed on 3 November 2009

^d^FAO Fishery and Aquaculture Country Profiles: Jamaica. http://www.fao.org/fishery/countrysector/FI-CP_JM/en

^e^CIPA Guía Indicativa: Nicaragua y el Sector Pesquero y Acuícola. Managua, Actualización en el 2007

^f^FAO Fishery and Aquaculture Country Profiles: Belize. http://www.fao.org/fishery/countrysector/FI-CP_BZ/en

^g^Based on HDI figures of 2010. Accessed on 15 November 2010

Table 2 highlights some significant differences between the three countries. Nicaragua is by far the largest producer of lobster. Jamaica, however, has the largest number of fishers, followed by Nicaragua and Belize. Lobster fishers in both Belize and Jamaica are known to operate within a multi-species fishery. This affects their annual income substantially as fishers are able to target other marine species when the lobster season is closed. Nicaragua and Jamaica also possess an industrial fishery whereas Belize has only a small-scale fishery.

**Table 2:** Fisheries characteristics in comparison

	Jamaica	Nicaragua	Belize
Lobster fishery volume export (tonnes. of tails per year)	460,000^a^	1,100,000	712,366^b^
# Fishers in total	20,000^c^	15,720	2,026
Lbs. of lobster per fisher	23^d^	70	352
Scale of fishery (small-or industrial?)	Small-scale and industrial	Small-scale and industrial	Small-scale
% of total catch per scale level	60 % small-scale and 40 % industrial^e^	50 % small-scale and 50 % industrial	100 % small-scale
Geartype	Wooden traps (industrial boats) Chicken wire traps (small-scale fishers) Hookah divers	SCUBA divers (industrial and small-scale) Wooden traps (industrial and small-scale)	Free-lung diving (direct for lobster or with use of casitas) Trapping (wooden traps)
Boats	3,874 small-scale boats 4 industrial lobster boats	78 industrial boats (51 trapping boats and 27 diving boats) 4,155 small-scale boats	652 small-scale boats
Length of trip (absence from home)	Daytrippers and fishers that leave for 7–10 days	Daytrippers or 20 days (industrial divers) or 45 days (industrial trappers)	Daytrippers (trappers) or 8–12 days (divers)
Single/multi species fishery	Multi-species (lobster, conch, fish)	Single species (only lobster)	Multi-species (lobster, conch, fish)

^a^FAO (2007)

^b^Average capture figure for 2003–2005 (FAO 2007)

^c^This is an estimate by the Jamaican government, only 14,000 fishers are registered officially as fisher (FAO 2007)

^d^Number in reality is higher as a large part of the lobster catch is designated for the national lobster market. These exact numbers are unknown and not calculated in this number

^e^Data is very inaccurate. Government of Jamaica has no precise data of landings of lobster

## The Lobster Fishery of Jamaica

Fishing in Jamaica has long been a source of both food and employment. The fishery makes a significant contribution to the Jamaican economy accounting for 0.4 % of the GDP and 7.5 % of the output of the Agriculture, Forestry and Fishing sector combined (Van Riel 2005) (see Table 1). The sector provides employment for many Jamaicans and contributes greatly to the food security and the alleviation of poverty. There are large fishing grounds on the southern shelf while the best fishing grounds of Jamaica are found at Pedro Bank, a large oceanic shelf 150 km to the southwest of Kingston.

Finfish, both in volume and value, is the most important marine export product; conch is second, followed by spiny lobster and shrimp. On average 700,000 tons of lobster tails are exported a year. However, this is only 15 percent of the total catch as the majority of the lobster catch goes to local hotels and restaurants (Venema 2004).

The current fishing industry is primarily small-scale. There are over 14,000 registered fishers, but estimates indicate that more than 20,000 Jamaicans are actually engaged in fishing (FAO 2007) (see Table 2). Fishers operate from some 186 fish landing beaches, including the offshore Pedro and Morant Cays. Fishers use a variety of gears varying from fish traps, beach seines, tangle and gill nets. They also engage in spearfishing, diving and some use of illegal explosives. Lobster fishers use traps, skin-diving equipment or hookah (diving with tubes from a small air compressor), while some are free lung divers.

Some fishers are day-trippers on the southern shelf but others live on the offshore cays more or less permanently. These migrant fishers are serviced by packer boats from the mainland, bringing supplies to the cays and produce to the mainland markets. Currently there are only four industrial boats in operation; the processing plant they are attached to is one of two plants that are allowed to export to the United States. Post-harvest structures varies according to the type of fishery. Small-scale fishers mostly sell their catch to beach vendors, middlemen or packer boats. The greater part is sold to hotels and restaurants and a smaller portion is destined for export (Venema 2004). Fishers are often dependent on middlemen for credit, fuel and gear. Only 6 % of the fishers in Jamaica belong to cooperatives (Kong 2005).

The government of Jamaica has established restrictions on the lobster fishery that resemble those in Belize and Nicaragua. However, the Fisheries Division is poorly staffed and lacks the necessary budget to enforce their regulations. In Jamaica 30 % of the total lobster sampled was under the minimum size while the inshore fishery of Jamaica is believed to be severely exploited (FAO 2007).

### Job Satisfaction in the Jamaican Lobster Fishery

Analysis of survey results show that the category nature scores highest, followed by social needs, basic needs, self-actualization and management. Mean values for all scores, except those for management, fall above the mid-point indicating general satisfaction with the fishery. The result for the category nature is striking as the fishery is generally believed to be highly overexploited. Nevertheless fishers express satisfaction with the state of landing sites and the level of stocks.

**Fig. 2:**
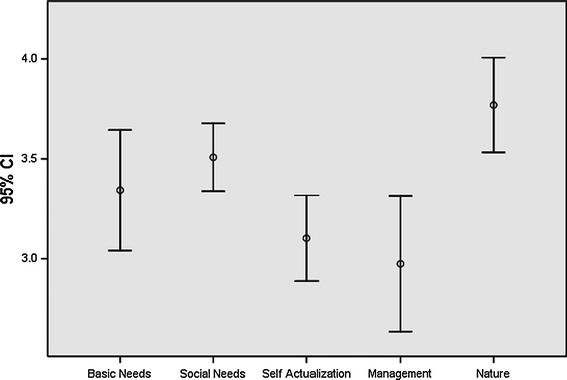
Mean values and confidence intervals for job satisfaction categories in Jamaica

The category social needs ranks second, closely followed by basic needs. Social needs relates to satisfaction in being one’s own boss and with the time fishers have available to spend with family and friends. The fact fishers are satisfied with basic needs is possibly related to the fact that the lobster fishery is part of a multi-species fishery in which fishers are able to target conch and finfish during the closed season for lobster. Management scores relatively low: fishers are clearly dissatisfied with the number of conflicts, with the way they are resolved, with the role of government officials and with the rules and regulations of the fishery (Fig. 2).

The general questions address willingness to change fishing type, leave the occupation of fishing or advise a young person to fish. In the Jamaican sample 62 % said they were unsure, 23 % said they would not and 15 % said they would change fishing type. A full 46 % said they would leave fishing for another occupation, while 39 % were unsure and 15 % said no. With regard to advising a young person to enter the occupation, almost all (85 %) said yes, 11 % said they were unsure and only 4 % said no. These responses indicate an ambivalence concerning the occupation, but the large percentage reporting that they would advise a young person to fish reflects positively on the desirability of fishing as an occupation.

We first examine these responses in relation to background social variables (Tables 3, 4, 5). For analytic purposes we grouped “no” and “undecided” together so that cell frequencies would be large enough to permit statistical analysis. Tables 3 and 4 indicate that there are no statistically significant relationships between willingness to change fishing type or change occupation altogether and the four social background variables. The only statistically significant difference is that those with more education (mean 12 years) are less likely than those with less education (mean 10.6 years) to advise a young person to enter the occupation of fishing (Table 5).

**Table 3:** Mean values of social background variables by willingness to change fishing type in Jamaica

	Change fishing type	N	Mean	SD	*t* value
Age	Yes	4	32.50	8.737	
	No/?	22	30.41	6.464	0.0.567
Education	Yes	4	11.00	2.000	
	No/?	22	10.82	1.468	0.217
Years fishing	Yes	4	10.00	6.733	
	No/?	22	9.45	5.316	0.182
Household size	Yes	4	2.50	1.915	
	No/?	22	2.45	1.819	0.046

* *p* < 0.05

**Table 4:** Mean values of social background variables by willingness to change occupation in Jamaica

	Change occupation	N	Mean	SD	*t* value
Age	No/?	14	31.64	8.317	
	Yes	12	29.67	4.228	0.743
Education	No/?	14	10.43	1.604	
	Yes	12	11.33	1.303	1.561
Years fishing	No/?	14	10.50	5.841	
	Yes	12	8.42	4.852	0.979
Household size	No/?	14	2.57	1.651	
	Yes	12	2.33	2.015	0.331

* *p* < 0.05

**Table 5:** Mean values of social background variables by willingness to advise a young person to fish

	Advise young to fish	N	Mean	SD	*t* value
Age	No/?	4	31.00	3.464	
	Yes	22	30.68	7.187	0.086
Education	No/?	4	12.00	0.000	
	Yes	22	10.64	1.560	4.101*
Years fishing	No/?	4	9.50	4.123	
	Yes	22	9.55	5.688	0.015
Household size	No/?	4	2.25	0.957	
	Yes	22	2.50	1.921	0.252

* *p* < 0.05 (calculated on the basis of equal variance not assumed)

Willingness to change is expected to be related to levels of job satisfaction—the higher the satisfaction the less willing a fisher should be to change fishing type or leave the occupation of fishing, and the more willing they should be to advise a young person to fish. Since we are predicting the direction of the relationship, one-tailed statistical tests of significance are used. Results indicate that those more satisfied with management are less likely to say they would change their fishing type (Table 6). This result is to be expected.

**Table 6:** Mean value of job satisfaction categories by willingness to change fishing type in Jamaica

	Change type	N	Mean	SD	*t* value
Basic needs	Yes	4	2.97727	0.459818	
	No/?	22	3.40909	0.776348	1.068
Social needs	Yes	4	3.50000	0.200000	
	No/?	22	3.50909	0.452411	0.039
Self actualization	Yes	4	2.91667	0.917928	
	No/?	22	3.13636	0.456106	0.468
Management	Yes	4	2.12500	0.315495	
	No/?	22	3.12879	0.814764	4.278*
Nature	Yes	4	3.75000	0.288675	
	No/?	22	3.77273	0.631085	0.070

*p < 0.05 (calculated on the basis of equal variance not assumed, 1-tailed test)

Fishermen scoring lower on the self actualization and nature categories of job satisfaction are more likely to report that they would leave the occupation of fishing (Table 7) and those more satisfied with the self actualization that fishing provides are more likely to encourage a young person to enter the occupation (Table 8). It should be noted that although many of the differences displayed in Tables 6, 7, and 8 are not statistically significant, they are all in the predicted direction.

**Table 7:** Mean value of job satisfaction categories by willingness to change occupation in Jamaica

	Change occupation	N	Mean	SD	*t* value
Basic needs	No/?	14	3.55195	0.939671	
	Yes	12	3.09848	0.318379	1.591
Social needs	No/?	14	3.61429	0.432981	
	Yes	12	3.38333	0.385730	1.425
Self actualization	No/?	14	3.30952	0.497245	
	Yes	12	2.86111	0.481125	2.327*
Management	No/?	14	3.21429	0.817617	
	Yes	12	2.69444	0.809768	1.623
Nature	No/?	14	4.00000	0.480384	
	Yes	12	3.50000	0.603023	2.353*

* *p* < 0.05 (1-tailed test)

**Table 8:** Mean value of job satisfaction categories by willingness to advise a young person to enter the occupation of fishing in Jamaica

	Advise young to fish	N	Mean	SD	*t* value
Basic needs	No/?	4	2.88636	0.465770	
	Yes	22	3.42562	0.764927	1.351
Social needs	No/?	4	3.35000	0.412311	
	Yes	22	3.53636	0.424876	0.810
Self actualization	No/?	4	2.41667	0.419435	
	Yes	22	3.22727	0.452931	3.322*
Management	No/?	4	2.54167	0.643702	
	Yes	22	3.05303	0.859719	1.126
Nature	No/?	4	3.75000	0.500000	
	Yes	22	3.77273	0.611930	0.070

* *p* < 0.05 (1-tailed test)

## The Lobster Fishery of Nicaragua

Lobster, shrimp and finfish occupy the 6th, 13th and 15th place in terms of the most valuable export products of Nicaragua (Rivera 2000), and the fishing sector contributes 1.5 % to total GDP (see Table 1). Currently, exports of lobster tails are around 1.1 million tons annually. Nearly all of the lobster is exported, mostly to the United States, as local demand is low. The best lobster fishing grounds of the country are around the Miskito Keys in the northern part of the Caribbean region of the country, and in the vicinity of Corn Island to the south (FAO 2001).

Until the 1950s, exploitation of the lobster resource on the Caribbean coast of Nicaragua was limited, owing mainly to the difficulty of access to international markets (World Bank 1999: 1). However, around this time foreign companies started obtaining contracts to fish for shrimp and other shellfish off the Caribbean Coast of Nicaragua and in the 1960s, industrial trapping began (Vilas 1989). The fleet grew tremendously until the civil war in 1979 brought the fishery to a near complete stop. In the period until 1989 there was a marked reduction in fishing effort on the coast (World Bank 1999: 8). With the end of the civil war, economic sanctions against Nicaragua were lifted and export of lobster tails started once again. The government granted a large number of fishing licenses to the industrial fleet. Since the 1990s lobster exports have increased significantly (Adpesca 2003).

Currently, there are 15,720 fishers active in Nicaragua (CIPA 2007). The majority of these (12,465 in number) are small-scale fishers while the rest are employed in the industrial fishery (see Table 2). There are four different fishing methods: small-scale trapping, small-scale diving, industrial trapping and industrial diving. Industrial trapping boats go out for 45 day trips and work with a crew of 13 to15 men (see Table 2). Nicaraguan small-scale divers and trappers generally only make day trips. Both small-scale as well as industrial fishers make use of very basic (if not primitive) diving equipment; for example, they lack depth meters and dive watches and use many tanks a day (10–14). Divers frequently suffer from decompression sickness and resulting injuries (e.g Acosta 2001, 2003; Nietschmann 1997; World Bank 1999).

There are many kinds of intermediaries between fishers and processing plants located in the region. First of all there the ‘bucket ladies’ who buy lobster on a small scale from fishers at landing sites. The more established middlemen, locally known as ‘acopios’, operate from fixed locations. Acopios are fully licensed and usually have large coolers full of ice and trucks to ferry fishing equipment, fuel, and ice for the fishers (Monnereau 2004). The majority of fishers sell their catch through middlemen although some also sell directly to processing plants. Approximately eight to ten processing plants are currently in operation on the Caribbean coast of Nicaragua. The processing plants own a majority of the industrial vessels operating in the region. Only a very limited number of the fishers belong to fishing cooperatives.

The government of Nicaragua has established restrictions and regulations regarding the lobster fishery. These include laws defining an annual closed season and prohibiting the catch of undersized and molting specimens, and berried females. However, enforcement of regulations is weak and the fisheries administration poorly staffed. Observers suggest that the Nicaraguan lobster fishery is highly overexploited (Ehrhardt 2006).

### Job Satisfaction in the Nicaraguan Lobster Fishery

**Fig. 3:**
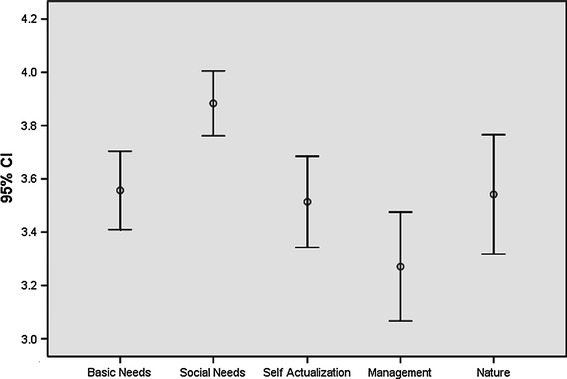
Mean values and confidence intervals of job satisfaction categories in Nicaragua

The Nicaraguan job satisfaction study show the following results per category (Fig. 3). Mean values for all scores fall above the mid-point of 3 indicating general satisfaction with the five categories of items. Social needs is the highest scoring category, followed by nature, basic needs, self-actualization and management.

Fishers score particularly high with regard to the category of social needs. Many of them do not feel dissatisfied with the time they are able to spend with friends and family. This connects to the fact that many of the respondents are day fishers who return to their homes every day.

Although older fishers are more likely to have higher scores on the self-actualization component (0.414) none of the other correlations are statistically significant (Table 9). This is to be expected. Fishers who enjoy fishing as a challenge or as an adventure and feel their job is worthwhile are more likely to stay in the fishery.

**Table 9:** Correlations between job satisfaction categories and selected social variables

	Basic needs	Social needs	Self actualize	Manage	Nature
Age	0.133	0.225	0.414*	0.205	0.286
Education	−0.197	0.127	−0.274	−0.074	−0.185
Years fishing	0.097	0.375	0.322	0.103	0.302
Household size	−0.275	0.056	0.219	0.245	0.193

* *p* < 0.05

In the Nicaraguan sample, only one fisher was willing to change fishing type or leave the occupation. The majority (69 %) reacted positively with regard to advising a young person to enter the occupation. Due to the extremely small number of fishers giving a positive response to the first two general questions, we are only able to conduct statistical analyses on responses concerning advising a young person to fish. However, the low level of responses to willingness to change type of fishing or leave the occupation and high numbers of those advising a young person to enter the occupation could relate to the fact the surveys are undertaken in an area where there are hardly any options other than lobster fishing. The economy of Corn Island depends highly on fishing, and alternatives are few.

Willingness to change is expected to be related to levels of job satisfaction—the higher the satisfaction the more willing they should be to advise a young person to fish. One-tailed statistical tests of significance are used to test this relationship. Table 10 indicates that those more satisfied with management are more likely to encourage a young person to enter the occupation. It should be noted that although most of the differences displayed in Table 10 are not statistically significant, all except the scores for Self Actualization are in the predicted direction.

**Table 10:** Mean value of job satisfaction categories by willingness to advise a young person to enter the occupation of fishing

	Advise young to fish	N	Mean	SD	*t* value
Basic needs	No	8	3.54545	0.266155	
	Yes	18	3.58081	0.373058	0.241
Social needs	No	8	3.77500	0.377018	
	Yes	16	3.93750	0.227669	1.322
Self actualization	No	8	3.70833	0.330344	
	Yes	18	3.44444	0.412231	1.592
Management	No	8	3.04167	0.671116	
	Yes	18	3.37963	0.327426	1.747*
Nature	No	8	3.37500	0.582482	
	Yes	18	3.66667	0.514496	1.282

* *p* < 0.05 (one-tailed test)

We examined the responses in relation to background social variables but the only statistically significant difference is that those with more education (mean 6.7 years) are more likely than those with less education (mean 3.9 years) to advise a young person to enter the occupation of fishing (Table 11). The relationships between willingness to advise a young person to fish and three social variables: marital status, fishing type, and crew position showed no statistically relevant outcome.

**Table 11:** Mean values of social background variables by willingness to advise a young person to fish

	Advise young to fish	N	Mean	SD	*t* value
Age	No	8	43.75	12.021	
	Yes	18	37.39	13.426	1.149
Education	No	8	3.88	1.356	
	Yes	18	6.67	2.326	3.143*
Years fishing	No	8	21.75	7.498	
	Yes	18	17.83	13.152	0.782
Household size	No	8	4.75	2.435	
	Yes	18	6.06	2.859	1.121

* *p* < 0.05

## The Lobster Fishery of Belize

Belize fisheries cater to domestic consumption as well as export markets and provide a large measure of livelihood and employment. The export of sea products is an important source of foreign exchange. The Belize barrier reef supports a variety of fisheries of which the spiny lobster, queen conch and pink shrimp are among the most important commercial species caught. During the last 40 years however it has been the spiny lobster fishery that has dominated the fishing industry. Over 500,000 tons is exported annually, mainly to the USA (FAO 2007) (see Table 1).

Between 1920 and 1960 the Belize fishery industry developed from a small-scale domestic fishery to an export fishery of lobster (*P. argus*), conch (*Strombus gigas*) and a variety of finfish for the lucrative US and Caribbean markets (Gillet 2003). During the 1960s the commercial fishery evolved from one dominated by foreign companies to locally owned cooperative organizations (Gillet 2003). Processing and freezing facilities were established in the 1950s in Belize City and in 1965 the government of Belize gave cooperatives exclusive rights over exports of fisheries products. There are now two fishing cooperatives with export licenses, and these cooperatives dominate the export market for lobster, conch and finfish products. Throughout the country there are several more fishing cooperatives. They, however, have do not have exporting facilities and work together with one of the two exporting processors in Belize City.

Credit unions, producer cooperatives and service cooperatives have been promoted in Belize for more than 55 years (McConney et al. 2003). Sixty percent of the lobster fishers in Belize are members of a fishing cooperative. McConney et al. consider the fishing cooperatives of Belize as the most successful of the cooperatives established in the country (McConney et al. 2003). The lobster fishery is the most important income earner in the highly developed small scale fishery in Belize. Belizean fishermen are able to get some of the best prices for lobster in the world (Huitric 2005). High export earnings strengthen the cooperatives economically, translating into political strength (Brown and Pomeroy 1999; McConney et al. 2003).

Fishers harvest lobster by skin-diving and traps. Divers in Belize work from sailboats, which carry around 10–13 divers, with each diver using a canoe to dive in the vicinity of the mother boat (see Table 2). Although there is a captain on board, which divers have to pay a daily fee, all divers are independent workers. Divers spend nine consecutive days out at sea and then take 2–3-day breaks.

Trap fishers in Belize make daytrips and go out every 2–3 days to haul their traps (locally referred to as ‘pots’). More often than not, fishers go out a few days a week and often combine fishing with other economic activities such as tourism. These fishers usually do not take ice to sea as the harvested lobsters are not killed until they reach shore.

The Belize government has established a set of restrictions and regulations regarding the lobster fishery. There are laws stipulating a closed season and prohibiting the catch of undersized and molting lobsters, and berried females. The level of overexploitation of the lobster resource in Belize is disputed (see FAO 2007; Huitric 2005).

### Job Satisfaction in the Belizean Lobster Fishery

**Fig. 4:**
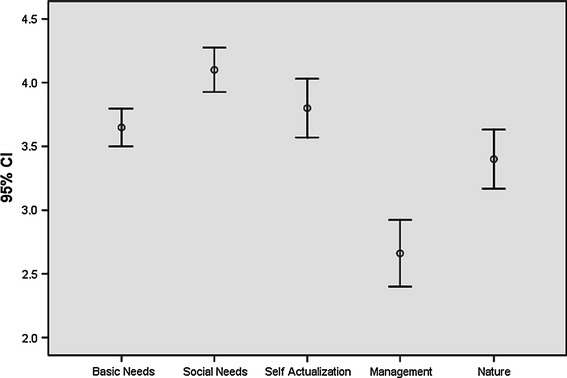
Mean values and confidence intervals for job satisfaction categories in Belize

The results of the job satisfaction study in Belize shows that fishers score high on the social needs category, followed by self actualization, basic needs, nature and management (Fig. 4). Most scores fall above the midpoint (of 3) demonstrating that fishers are satisfied on most counts except for management.

The high scores on social needs and self-actualization could relate to the fishers’ autonomous position in the lobster fishery. They are often independent workers. Middlemen are present but fishers mostly sell to the cooperative to which they belong. None of the fishers spend more time away from their family than 9 days and if a fisher is not a captain he will at times skip a week to spend more time with the family. In addition, fishers work no more than approximately 8 h a day. In the evenings they listen to the radio and play games like domino. Day trap fishers are home every day with their family and friends and therefore do not miss spending time with them.

The basic needs category scores high as well. This is related to the fact Belize is a multi-species fishery. During the closed season for lobster, fishers go out for finfish or conch; during the closed season for conch they target lobster and finfish. Because the fishery is organized around two fishing cooperatives which hold exclusive rights over seafood exports, fishers are able to reap some of the highest fishing incomes in the region (Huitric 2005). All profits made by the fishing cooperatives flow back to the fishers at the end of the fiscal year.

However, analyses of the general questions show that 32 % of the Belizean sample states they would be willing to leave their current fishing type, 61 % say they would leave fishing for another occupation and only 29 % point out that they would advise a young person to enter the occupation of fishing. In view of the high values recorded in Fig. 4 these results are surprising.

Results show that there are no significant relationships between the background variables (age, marriage, education level and fishing experience) and willingness to change occupation or fishing type (Tables 12, 13). However, results do indicate that older fishers and those with more fishing experience are less willing to leave the occupation than younger or less experienced fishers.

**Table 12:** Mean values of social background variables by willingness to change occupation

	Change occupation	N	Mean	SD	*t* value
Age	No	12	54.42	17.159	
	Yes	19	41.11	10.236	2.428*
Education	No	12	5.25	2.179	
	Yes	19	6.00	2.603	0.830
Years fishing	No	12	34.33	15.388	
	Yes	19	22.05	8.376	2.537*
Household size	No	12	2.58	1.505	
	Yes	19	3.26	2.207	0.936

* *p* < 0.05

**Table 13:** Mean value of job satisfaction categories by willingness to change fishing type in Belize

	Change fishing type	N	Mean	SD	*t* value
Basic needs	Yes	10	3.60909	0.540465	
	No	21	3.62338	0.372933	0.086
Social needs	Yes	9	3.84444	0.572519	
	No	21	4.20952	0.376702	2.075*
Self actualization	Yes	10	3.90000	0.737865	
	No	21	3.73016	0.553966	0.717
Management	Yes	10	2.70000	0.723503	
	No	21	2.64286	0.687761	0.213
Nature	Yes	10	3.20000	1.005540	
	No	21	3.38095	0.610425	0.623

* *p* < 0.05 (1-tailed test)

Willingness to change could also be expected to correlate with levels of job satisfaction—the higher the satisfaction the less willing a fisher should be to change fishing type or leave the occupation of fishing, and the more willing he should be to advise a young person to fish. Mean values on job satisfaction categories in relation to responses to these questions are examined in Tables 13, 14, and 15.

**Table 14:** Mean value of job satisfaction categories by willingness to change occupation in Belize

	Change occupation	N	Mean	SD	*t* value
Basic needs	Yes	19	3.52153	0.477061	1.649
	No	12	3.77273	0.278182	
Social needs	Yes	18	4.03333	0.445896	0.958
	No	12	4.20000	0.497265	
Self actualization	Yes	19	3.71930	0.631119	0.746
	No	12	3.88889	0.591750	
Management	Yes	19	2.69298	0.583368	0.318
	No	12	2.61111	0.853789	
Nature	Yes	19	3.21053	0.751217	1.052
	No	12	3.50000	0.738549

**Table 15:** Mean value of job satisfaction categories by willingness to advise a young person to enter the occupation of fishing in Belize

	Advise young to fish	N	Mean	SD	*t* value
Basic needs	Yes	9	3.79798	0.382407	1.536
	No	22	3.54545	0.427323	
Social needs	Yes	9	4.00000	0.100000	0.764
	No	21	4.14286	0.551880	
Self actualization	Yes	9	4.03704	0.538631	1.497
	No	22	3.68182	0.621291	
Management	Yes	9	2.75926	0.707652	0.501
	No	22	2.62121	0.692327	
Nature	Yes	9	3.72222	0.666667	1.996*
	No	22	3.15909	0.730074	

* *p* < 0.05 (1-tailed test)

Since this paper is predicting the direction of the relationship, one-tailed statistical tests of significance are used. The analysis presented in Table 13 indicates that those who say they are unwilling to change fishing type are likely to score higher on the social needs job satisfaction category. These fishers enjoy being out at sea, enjoy being their own master and are not dissatisfied with the time they have available to spend with friends and family. It is therefore not surprising they are less willing to change occupation. The mean values of job satisfaction categories by willingness to change occupation show no statistically significant results (Table 14). Finally, Table 15 indicates that those who are willing to advise a young person to enter the occupation of fishing are more likely to score higher on the nature category of job satisfaction. As this category holds the important question on the levels of (over)exploitation of the resource this relationship could be expected. Fishers who are more satisfied with the level of stocks are more likely to advise a young person to enter the fishery. All other differences, except one, are in the predicted direction, but not statistically significant.

## Job Satisfaction Across the Caribbean Countries

**Fig. 5:**
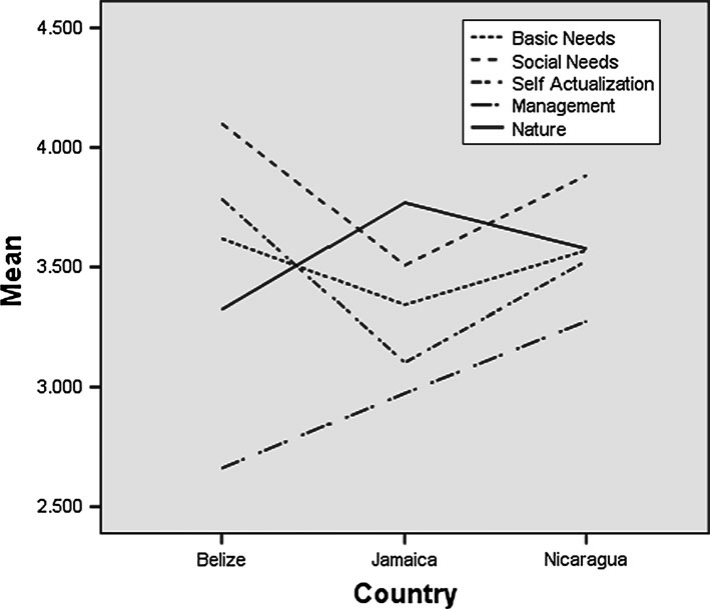
Means for job satisfaction categories across the different countries

The three countries show distinct patterns in terms of job satisfaction outcome. Mean values for all scores, except management, fall above the mid-point of 3 indicating general satisfaction with the other four categories of job satisfaction. Table 16 shows the mean values by country per job satisfaction category. This table indicates that there are statistically significant differences in levels of satisfaction for all categories except basic needs across the three countries. These results are also depicted in Fig. 5.

**Table 16:** Means per question per country

	SAFETY	PREDEARN	EARNINGS	MENTALPR	CLEAN	HOURFISH	HEALTH	FATIGUE	TIMEGND
Jamaica	4.346	3.231	3.654	2.692	3.423	3.308	2.577	2.654	3.346
Nicaragua	3.538	3.577	3.423	3.385	4.000	3.654	3.808	3.615	3.808
Belize	3.516	3.323	3.484	3.419	3.935	3.871	3.774	2.935	4.516

	FOODSECU	CATCHLVL	TIMESEA	AWAYHOME	OWNBOSS	COMMUNIT	RECREATE	CHALLENG	ADVENTUR
Jamaica	4.154	3.385	3.423	3.192	4.692	3.538	2.692	2.962	3.423
Nicaragua	2.885	3.577	3.800	3.885	4.154	3.846	3.720	3.731	3.731
Belize	4.065	2.968	4.226	3.645	4.467	4.258	3.903	3.613	3.806

	WORTHWH	CONFFISH	CONFRESO	MANAGEM	GOVPERF	RULESREG	INFLMGMT	PORTLAND	FISHSTOK
Jamaica	2.923	2.615	2.808	3.154	2.615	3.385	3.269	4.077	3.462
Nicaragua	3.115	2.885	2.846	3.115	3.308	3.846	3.654	3.808	3.346
Belize	3.935	2.903	2.677	2.516	2.387	2.968	2.516	4.000	2.645

Belize and Nicaragua show a relatively similar ranking pattern except for the category of management. Belize scores very low on management whereas Nicaragua shows higher results. The reverse would actually be expected. Further research is needed to explain this discrepancy. The graphs show clearly that in all countries the category management scores lowest. Within this category fishers are especially dissatisfied with the performance of government officials (Table 17). Nonetheless, the questions on conflicts and conflict resolution also score exceptionally low across all countries (Table 17). This reflects the fact fishers perceive the presence of conflicts in their fishery and the lack of resolutions for these.

**Table 17:** Job satisfaction category mean values by country

	N	Mean	SD	F value	
Basic needs	Belize	31	3.61877	0.424731	
	Jamaica	26	3.34266	0.746256	
	Nicaragua	26	3.56993	0.338746	2.133
Social needs	Belize	30	4.10000	0.466092	
	Jamaica	26	3.50769	0.420403	
	Nicaragua	24	3.88333	0.288424	15.068*
Self actualization	Belize	31	3.78495	0.611909	
	Jamaica	26	3.10256	0.531407	
	Nicaragua	26	3.52564	0.401918	11.880*
Management	Belize	31	2.66129	0.687836	
	Jamaica	26	2.97436	0.840228	
	Nicaragua	26	3.27564	0.473620	5.721*
Nature	Belize	31	3.32258	0.747757	
	Jamaica	26	3.76923	0.587040	
	Nicaragua	26	3.57692	0.542076	3.503*

* *p* < 0.05

Nicaragua also scores higher on the nature category than Belize. Jamaica, however, scores highest. Although the exact levels of overexploitation are unknown in Jamaica they are believed to be relatively high in comparison to Belize and at least equal to that of Nicaragua. According to the FAO (2007) the lobster fishery in Nicaragua and Jamaica are both overexploited while the lobster fishery is regarded as fully-exploited.

This could be related to the fact the national tourist industry is also a large buyer of lobsters. Even though Jamaican fishers might be fishing undersized lobster they still have a market whereas this market is much smaller in Nicaragua and to a lesser extent in Belize.

The categories of basic needs, social needs, and self-actualization scores are very low in Jamaica in comparison to the other two countries. Although fishers are relatively positive towards the level of exploitation they are much less satisfied with the extent to which their basic needs, social needs and self-actualization needs are met. Additional research is required to explain these results.

Table 18 shows the correlation between job satisfaction categories and selected social variables. As years of fishing experience increase so does level of satisfaction on the basic needs, social needs and self actualization categories. In contrast, as education level increases, levels of satisfaction with the social needs and self actualization categories decrease. Finally, the results show that the older a fisher is the more likely he will be satisfied on the social needs category.

**Table 18:** Correlations between job satisfaction categories and selected social variables

	Basic needs	Social needs	Self actual	Manage	Nature
Age	0.146	0.349*	0.210	−0.149	−0.070
Education	−0.134	−0.391*	−0.416*	−0.009	0.120
Years fishing	0.243*	0.444*	0.289*	−0.155	−0.080
Household size	0.050	0.031	0.112	0.188	0.037

There are no statistically significant differences between married and single fishers on the five job satisfaction categories (Table 19). Fishing type, however, is related to satisfaction with management—trap fishers are less satisfied than dive fishers (Table 20). Finally, captains are more satisfied than crew members with the basic needs, social needs and self actualization categories of job satisfaction (Table 21). They are less satisfied with the management category. This could be related to the fact they expect more from the government than crewmembers do.

**Table 19:** Job satisfaction category mean values by marital status

	Married	N	Mean	SD	*t* value
Basic needs	Single	29	3.49843	0.733182	
	Married	54	3.52694	0.396216	0.230
Social needs	Single	29	3.71724	0.429342	
	Married	51	3.91373	0.483744	1.817
Self actualization	Single	29	3.33333	0.549170	
	Married	54	3.57407	0.605415	1.783
Management	Single	29	3.06897	0.725824	
	Married	54	2.88889	0.719597	1.084
Nature	Single	29	3.65517	0.669534	
	Married	54	3.48148	0.651290	1.147

* *p* < 0.05

**Table 20:** Job satisfaction category mean values by fishing type

	Metier	N	Mean	SD	*t* value
Basic needs	Trap fisher	49	3.57514	0.603989	
	Dive fisher	30	3.39697	0.414529	1.422
Social needs	Trap fisher	48	3.88750	0.468417	
	Dive fisher	28	3.77143	0.506884	1.011
Self actualization	Trap fisher	49	3.52381	0.581346	
	Dive fisher	30	3.38889	0.631748	0.969
Management	Trap fisher	49	2.81633	0.736958	
	Dive fisher	30	3.18889	0.694441	2.228*
Nature	Trap fisher	49	3.60204	0.568070	
	Dive fisher	30	3.51667	0.793110	**0.514**

* *p* < 0.05; Bold = equal variance not assumed

**Table 21:** Job satisfaction category mean values by position

	Position	N	Mean	SD	*t* value
Basic needs	Crew	46	3.39130	0.427206	
	Captain	37	3.67322	0.613387	2.463*
Social needs	Crew	44	3.68636	0.444915	
	Captain	36	4.03333	0.436872	3.498*
Self actualization	Crew	46	3.34058	0.642100	
	Captain	37	3.67568	0.474579	2.645*
Management	Crew	46	3.13043	0.640132	
	Captain	37	2.72973	0.765127	2.598*
Nature	Crew	46	3.54348	0.751488	
	Captain	37	3.54054	0.532008	**0.021**

* *p* < 0.05; Bold = equal variance not assumed

### Willingness to Change

A little less than half of the fishers, 47 % in all the countries said they would be willing to change fishing type. A higher number of fishers expressed that they would be willing to leave the occupation, a majority said they would advise a young person to enter the occupation of fishing.

These responses indicate ambivalence. The analysis of the distributions in Tables 22, 23, and 24 indicates that the country differences in response to all three questions are statistically significant. Overall, the Jamaican fishers seem to be more positive concerning the occupation of fishing. This could be related to their higher satisfaction with the nature category. This shows higher confidence in the future exploitation of fish stocks and therefore a greater tendency to advise young people to enter the fishery. Nicaraguan fishers, especially, indicated they would be willing to change occupation—96 % indicated they would. This could be related to the fact the surveys were carried out in a place where there are no other options. Corn Islands’ main economic pillar is the lobster fishery.

**Table 22:** Percent distribution of willingness to change occupation by country

	Belize	Jamaica	Nicaragua	Total	N
No	38.710	53.846	3.846	32.530	27.000
Yes	61.290	46.154	96.154	67.470	56.000
N	31.000	26.000	26.000		83.000

χ^2^ = 15.669; *df* = 2; *p* < 0.05

**Table 23:** Percent distribution of willingness to change fishing type by country

	Belize	Jamaica	Nicaragua	Total	N
Yes	32	15	9	47	39
No	68	85	4	53	44
N	31	26.000	26		83

χ^2^ = 38.357; *df* = 2; *p* < 0.05

**Table 24:** Percent distribution of willingness to advise young to fish by country

	Belize	Jamaica	Nicaragua	Total	N
No	70.968	15.385	30.769	40.964	34.000
Yes	29.032	84.615	69.231	59.036	49.000
N	31.000	26.000	26.000		83.000

χ^2^ = 19.692; *df* = 2; *p* < 0.05

Additional data shows that fishers with more education and smaller household sizes are less in favor of changing fishing type. In addition fishers with larger households are more likely to say they would leave the occupation of fishing. This is possibly in relation to their greater need for stable and predictable income. Finally, fishers who would advise a young person to enter the occupation are younger, have more years of education, and have fished fewer years. These fishers have obviously only recently made this decision themselves. These results are therefore to be expected. Older fishers have seen the resource decline over the last decades which would probably make them less inclined to advise a young person to enter the fishery.

Thirty-one percent of the single fishers versus 56 % of married fishers say they would change fishing type, 44 % of the trap fishers and 40 % of the dive fishers report they would change fishing type—a difference that is not statistically significant. Finally, 43 % of the captains and 50 % of the crew say they would change type, a difference that is also not statistically significant.

Willingness to change is expected to be related to levels of job satisfaction—the higher the satisfaction the less willing a fisher should be to change fishing type or leave the occupation of fishing, and the more willing they should be to advise a young person to fish. Mean values on job satisfaction categories in relation to responses to these questions are examined in Tables 25, 26, and 27. Since we are predicting the direction of the relationship, one-tailed statistical tests of significance are again used.

**Table 25:** Mean value of job satisfaction categories by willingness to change fishing type

	Change type	N	Mean	SD	*t* value
Basic needs	Yes	39	3.50816	0.435844	
	No	44	3.52479	0.613094	0.141
Social needs	Yes	36	3.82778	0.382929	
	No	44	3.85455	0.537632	0.259
Self actualization	Yes	39	3.54701	0.609319	
	No	44	3.43939	0.583019	0.822
Management	Yes	39	3.00000	0.651135	
	No	44	2.90909	0.785408	0.570
Nature	Yes	39	3.48718	0.683328	
	No	44	3.59091	0.640428	0.714

**Table 26:** Mean value of job satisfaction categories by willingness to change occupation

	Change occupation	N	Mean	SD	*t* value
Basic needs	No	27	3.63973	0.701230	
	Yes	56	3.45779	0.426389	1.464
Social needs	No	27	3.88889	0.533013	
	Yes	53	3.81887	0.440741	0.625
Self actualization	No	27	3.58025	0.596232	
	Yes	56	3.44643	0.593830	0.961
Management	No	27	2.97531	0.880345	
	Yes	56	2.94048	0.641517	**0.183**
Nature	No	27	3.77778	0.640513	
	Yes	56	3.42857	0.642641	2.322*

* *p* < 0.05; Bold = equal variance not assumed

**Table 27:** Mean value of job satisfaction categories by willingness to advise a young person to enter the occupation of fishing

	Advise young to fish	N	Mean	SD	*t* value
Basic needs	No	34	3.46791	0.444310	
	Yes	49	3.55102	0.590445	0.695
Social needs	No	33	3.95758	0.560708	
	Yes	47	3.76170	0.383663	1.741*
Self actualization	No	34	3.53922	0.676765	
	Yes	49	3.45578	0.534434	0.627
Management	No	34	2.71078	0.688174	
	Yes	49	3.11905	0.704647	2.621*
Nature	No	34	3.27941	0.687454	
	Yes	49	3.72449	0.577902	3.191*

* *p* < 0.05; Bold = equal variance not assumed

Table 24 indicates that willingness to change fishing type is not statistically significantly related to scores on any of the job satisfaction categories. Fishers who are less satisfied with the level of stocks are more willing to leave the occupation of fishing (Table 25). Fishers who would advise a young person to enter the occupation of fishing score lower on the social needs category (opposite the predicted direction) and higher on the management and nature categories (Table 27).

## Discussion and Conclusion

It is clear from the results that in all three countries, fishers like their occupation. The lobster fishers show a general satisfaction with their occupation. They highly value their occupation and despite an overall declining resource a majority said they would advise a young person to enter the occupation of fishing.

As the resource is declining throughout the area, a question concerning characteristics of the occupation of fishing that make it attractive arises. The data show that the categories basic needs, social needs and self-actualization score high (especially in Nicaragua and Belize). Consequently, if one wishes to provide an alternative occupation to fishers, it will need to be attractive to them on those accounts.

Across all three countries fishers are least satisfied with management. This is shared in all job satisfaction studies carried out, where fishers targeted government officers as the object of their greatest dissatisfaction (Bavinck and Monnereau 2007: 148). Although the management category scores are higher in Nicaragua, the category still scores lower than all other categories. These results show that good communication and cooperation between fishers and government at all levels is often lacking. Fishers are very dissatisfied with government officials–a problem that could be improved. However, results also indicated fishers are very dissatisfied with both the level of conflict as well as conflict resolution in fisheries. The survey is does not indicate what types of conflicts are implied by the fishers: between different gear types (e.g. divers vs. trappers), different ethnic groups (e.g. in Nicaragua between Miskito’s and creole fishers), scales (small-scale versus industrial) or between fishers and non-fishing activities (e.g. fishers and tourist activities). In order to improve management of these fisheries these aspects of conflict resolution should be explored. However, quantitative data gathering is necessary in order to find the exact types of conflict that shape the satisfaction of fishers with their occupation. Decreasing the level of conflicts could therefore greatly improve the functioning of the fishery.
